# Adversarial Homicide-Suicide Perpetrated by Domestic Helper: A Case Report From Nepal

**DOI:** 10.7759/cureus.46847

**Published:** 2023-10-11

**Authors:** Kaschev Shrestha, Alok Atreya

**Affiliations:** 1 Department of Forensic Medicine, College of Medical Sciences, Bharatpur, NPL; 2 Department of Forensic Medicine, Lumbini Medical College, Palpa, NPL

**Keywords:** violence, strangulation, murder-suicide, hanging, forensic medicine, delusion, adolescent

## Abstract

The majority of literature on homicide-suicide addresses the fact that victims are predominantly female, and offenders are typically adult males (older than the victims) who share a familial, marital, or consortial relationship with them. The probability of fatalities involving murder-suicides in the bedrooms of middle-class households is higher. We present a case where an adolescent domestic helper strangled his landlady, twice his age, only to commit suicide by hanging thereafter. We go on to discuss homicide-suicide by servants outside the consortial relationship and the possible reasons for it in the Nepalese context.

## Introduction

A homicide-suicide is a form of lethal violence in which a person kills one or more individuals without their consent and then takes their own life shortly thereafter [1,2]. Although the majority of the time the perpetrator commits suicide immediately after the victim is killed, there have been reports of suicides occurring up to three months later [3,4]. Homicide-suicide, according to Berman, is a subset of “dyadic deaths,” defined as two people dying together in a related sequence [5]. Whereas, homicide-suicide involves the non-consensual killing of others prior to suicide, “pact suicide,” refers to situations where two or more individuals jointly plan and willfully agree to end their lives together [5,6].

A key distinction between dyadic death pacts and homicide-suicides is consent - in pact suicides, both parties willingly consent to the arrangement, while the victim is an unwilling participant in a homicide-suicide [6]. For an event to be classified as a homicide-suicide rather than a dyadic death pact, there must be clear evidence of a chronological sequence showing homicide preceding suicide and a motivational link between the homicide and subsequent suicide [7].

Homicide-suicides differ from pact suicides in that one person chooses to end another’s life along with their own, without mutual planning or agreement. After committing the homicide(s), the perpetrator takes their own life, typically within minutes to days of the initial killings [8]. This introduces an element of coercion not present in jointly planned dyadic death pacts. The evidence required to confirm a true homicide-suicide rather than a suicide pact includes establishing a clear order of events and motive linking the acts of homicide and subsequent suicide.

Despite the fact that homicide-suicide is a rare occurrence, with an incidence of 0.2 to 0.3 per 100,000 population, it receives a great deal of media attention due to its uniqueness, giving the impression that it is happening more frequently [8,9]. The majority of the literature on homicide-suicide addresses the fact that victims are predominantly female and that offenders are typically adult males (older than the victims) who share a familial, marital, or consortial relationship with them [3,10,11]. In homicide-suicide cases involving children, however, the perpetrator can be either male or female who shares a filial relationship with the victim and commits filicide [1,4,11]. The likelihood of fatalities involving murder-suicides in the bedrooms of middle-class households is higher [4]. We present a typical case from Nepal, where the victim was strangled on the kitchen floor by a male perpetrator who was her domestic helper and half her age.

In the current case, we present from Nepal, the evidence points to a homicide-suicide rather than a dyadic death pact, based on the following: 1) lack of any evidence of a mutual plan or consent by the victim, 2) clear chronological order of homicide preceding suicide, and 3) motivation of perpetrator apparent in strangling an unwilling victim preceding his own suicide. This evidence confirms the key criteria differentiating homicide-suicide from dyadic deaths [8].

## Case presentation

After returning home from a morning walk, the landlord discovered that his domestic helper was hanging from the balcony railing inside his house. He rushed into the kitchen to find his wife in a pool of blood on the floor. He then immediately took her to a nearby hospital for treatment, and the police were notified. However, the female victim was unable to survive and died as a result of her injuries. A police inquest was held and her body was sent to the mortuary along with a request from the police for forensic pathologists to visit the crime scene.

Crime scene

**Figure 1:**
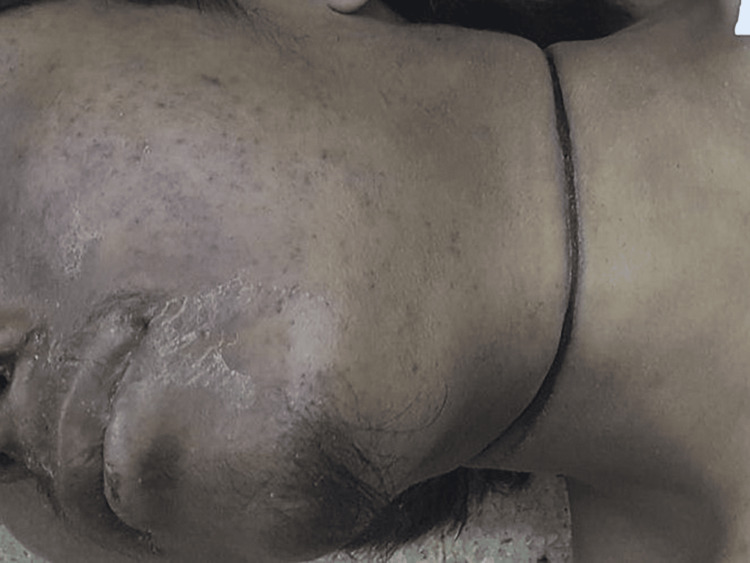
Photograph of the perpetrator The photograph depicts a furrowed ligature mark encircling the neck of the homicide-suicide perpetrator who died by hanging himself with a nylon rope, with dry salivary stains at the left angle of his mouth.

At the crime scene, a body was seen lying on the ground in a supine position. The ligature was cut to lower the body, according to the police. A green nylon rope with its other end torn was tied to the floor upstairs railing just above the body. Both hands’ palmer surfaces were covered in dried blood stains. The lower limb had patches of post-mortem lividity that blanched on pressure, rigor mortis was evident on the upper limb, and both eyes had tache noire. There were dry salivary stains present dribbling from the left angle of the mouth and extending up to the left side of the chin (Figure 1). The body was then sent to the mortuary for further examination.

On entering the kitchen, blood stains were noticed on the floor where the victim was allegedly recovered. There were horizontal blood splatters about 12 inches above the base of the refrigerator, adjacent to the area where there was a smear of blood on the floor. A nylon rope lay on the floor, one end tied to the leg of a chair. A plastic stool had been shattered into pieces, one of which had dried bloodstains.

Autopsy of the wife of landlord

**Figure 2:**
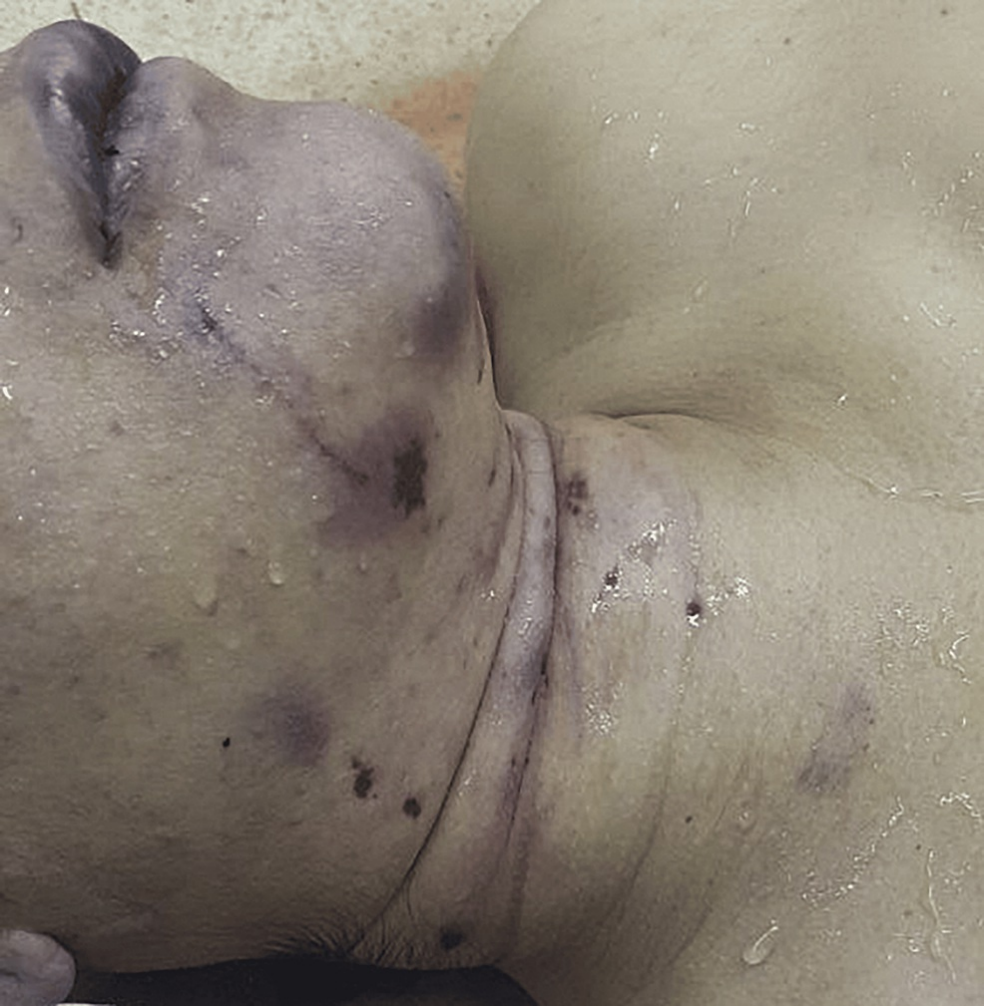
Photograph of the neck region of the victim The photograph shows a ligature mark along with several blunt force injuries.

The body brought to the mortuary for autopsy was a middle-aged woman with a smear of blood on her face and clothes. Rigor mortis was complete and post-mortem lividity was present over the posterior aspect of the body which was purplish and blanched under pressure. A reddish ligature mark with several horizontal and erratically spaced reddish abrasions, each measuring an average of 3 cm in width, is visible above, below, and on both sides of the thyroid cartilage (Figure 2). The ligature mark was incomplete and not present on the posterior aspect of the neck. There was evidence of multiple blunt force injuries over the face in the form of lacerations, abrasions, and contusions. An area approximately 8 cm by 6 cm, on the forehead, at the anterior midline, and 2.5 cm from the top of the head, had more than 10 reddish contusions of varying sizes from a few mm to 1 cm by 3 cm. Bilateral bluish periorbital contusions were present. Contused abrasions present around the mouth and lacerations present over the inner aspect of the lips suggested smothering. Multiple reddish linear contusions, ranging from 1 cm x 1 cm to 3 cm x 0.5 were present on the anterior aspect of the lower third of both forearms suggesting the restraint of the victim.

Internally, multiple contusions were present in the frontal, parietal, bilateral temporal, and occipital regions of the reflected scalp. The skull and brain were free of injury. Contusions were present in the upper third, middle third of both sternocleidomastoid muscles, and the upper third of both sternothyroid muscles. There were contusions present on both sides of the thyrohyoid muscle. The hyoid bone was free of fractures. There was no evidence of laryngeal edema. Contusions were found over the intercostal muscles on the right side of the chest wall. There was a fracture over the right 7th and right 8th rib. There was a contusion present over the hilum of the left lung. In the right auricle, there was an evident laceration with a surrounding contusion and 100 ml of blood was drawn in the pericardial sac. There were no signs of forceful sexual intercourse. Swabs collected from the victim’s vagina did not detect any sperm. The cause of death was opined to be asphyxia due to ligature strangulation.

Autopsy of the perpetrator

The body was that of a young adult male with a reddish brown parchmentized and deeply furrowed ligature mark with an average width of 0.5 cm present on the neck. The mark was located immediately above the thyroid cartilage, inclined upward and backward on the right side, horizontally backward on the left up to the lateral aspect of the neck, then heading upward and backward. The ligature mark on both sides disappeared on the posterior hairline. Internally, there was laryngeal edema with intact laryngeal cartilage. There were no soft tissue hemorrhages and the hyoid bone was free of fractures. Other findings were not remarkable. Swabs were collected from the glans penis and the shaft of the penis of the deceased, which did not reveal any vaginal epithelial cells. The cause of death was opined to be asphyxia due to hanging.

## Discussion

The majority of murder-suicides involve a man killing his spouse or consortial partner, such as his wife, girlfriend, ex-wife, or ex-girlfriend [8]. Spousal or consortial type of homicide-suicide is more commonly reported in which the female’s rejection of her boyfriend, followed by her threat of withdrawal or estrangement, is a common trigger event, the perpetrator cannot withstand rejection or separation [3,12]. In contrast, when the wife is the perpetrator and the husband is the victim, it is less likely that suicide will follow the homicide [13]. The other type of homicide-suicide is familial type in which parents or older family members murder children and later commit suicide [3]. The third type of murder-suicide is extrafamilial, which is the least common [3].

Works of literature suggest that most homicide-suicides are planned events, by the perpetrator suffering from depression or paranoia, with involvement of alcohol and drugs [3,13,14]. In contrast, in the present case, it was an extrafamilial type of murder-suicide, the perpetrator lived under the same roof as a domestic help, there was no alcohol and drug involvement, and there was no sexual intent. It was less likely that the homicide-suicide in the present case was a premeditated event and there was no history of the perpetrator suffering from mental illness. Outside-the-family murder-suicides are usually committed by a resentful employee or former employee seeking vengeance for actual or imagined insults, injury, or mistreatment [4]. These “adversarial homicide-suicides” are caused by persecutory delusions caused by not being paid for services rendered or receiving a pay raise, however, in the present case it was less likely [4].

In the context of Nepal, where many domestic helpers are impoverished individuals, often children of tenants working for wealthier landlords, it is vital to consider the challenging living and working conditions they endure [15]. These circumstances can include poverty, limited education, and positions of powerlessness, which may lead to exploitation, abuse, or mistreatment [15]. The poor servants are enslaved to resist any form of physical, mental, or sexual abuse [15]. The actual reason for murder in the present case could not be ascertained which requires a verbal autopsy of other servants, the landlord, and the family of the perpetrator. However, an educated guess can be made that the perpetrator attempted to murder her first with blunt force trauma and finally strangling her. While the specific trigger for the violent actions in this case cannot be definitively determined, it is plausible that the perpetrator’s rage against the landlady stemmed from some form of abuse or mistreatment. Unfortunately, due to his vulnerable position, he may have felt unable to voice his grievances or seek justice. It is important to note that the perpetrator’s actions were not justified, but understanding the broader context can shed light on the complex factors contributing to such tragic events. Due to his youth and hot blood, he could have chosen to retaliate rather than suffer as a victim. There was no way that the perpetrator could hide or flee without being caught. The perpetrator might have realized the consequences of his brutal act, so rather than being punished by the law, he chose to take away his life by himself.

## Conclusions

This case illustrates an uncommon extrafamilial homicide-suicide event perpetrated by a young domestic helper against his female employer. The perpetrator was an adolescent male domestic servant who strangled his middle-aged female employer to death and then committed suicide by hanging. The exact trigger that led to this tragic event remains unknown. However, the inequality in power dynamics, insecure work environment, and lack of legal protections for domestic help in Nepal likely contributed as risk factors. Extrafamilial homicide-suicides by domestic help are rare events that have not been well studied. Further research is needed to understand the motivations behind such acts, in order to prevent similar tragedies in the future. Adequate legal protections and recourse against abuse should be established for domestic workers. Addressing root causes like poverty, lack of education, and power imbalances is crucial. This case highlights the need for social reforms to improve the lives of domestic helpers in Nepal and other developing countries.
